# “I do think that accessibility is a really major thing that has come [out] of [the] pandemic”: The lived experiences of resilience and health-related quality of life among a diverse sample of graduate students during the COVID-19 pandemic

**DOI:** 10.1371/journal.pone.0309171

**Published:** 2024-08-20

**Authors:** Katie J. Shillington, Shauna M. Burke, Tara Mantler, Jennifer D. Irwin

**Affiliations:** 1 Health and Rehabilitation Sciences, Faculty of Health Sciences, The University of Western Ontario, London, Ontario, Canada; 2 Department of Neurobiology, University of California San Diego, San Diego, California, United States of America; 3 Center for Empathy and Social Justice in Human Health, T. Denny Sanford Institute for Empathy and Compassion, University of California San Diego, San Diego, California, United States of America; 4 Department of Psychology, Faculty of Science, Wilfrid Laurier University, Waterloo, Ontario, Canada; 5 School of Health Studies, Faculty of Health Sciences, The University of Western Ontario, London, Ontario, Canada; 6 Children’s Health Research Institute, London, Ontario, Canada; Lamar University, UNITED STATES OF AMERICA

## Abstract

The COVID-19 pandemic has negatively impacted the mental health and wellbeing of post-secondary students. Resilience has been found to serve as a protective factor against mental distress among students during the pandemic. Despite the plethora of research that exists on post-secondary students during this crisis, most studies exploring students’ health and resilience are quantitative and lack diversity. To date, the lived experiences of health-related quality of life (HRQOL) and resilience among graduate students representing diversity in age, gender, ethnicity, parental status, university, degree, and faculty during the COVID-19 pandemic remain unknown. As a part of a larger study, the purpose of this qualitative paper was to understand the lived experiences of resilience and HRQOL among a diverse sample of graduate students approximately 18 months into the COVID-19 pandemic in Ontario, Canada. A total of 14 students participated in semi-structured interviews exploring HRQOL domains, factors that supported/undermined participants’ resilience, challenges/barriers to being resilient, and participants’ inner strength. Thematic analysis revealed 5 themes: (1) cultural influences on resilience; (2) the role of privilege/power in shaping resilience; (3) how life stage and past experiences support resilience; (4) how the COVID-19 pandemic has undermined the resilience of equity-deserving groups; and (5) the role of disability/chronic pain. This work presents a unique dichotomy between how the COVID-19 pandemic has disrupted the lives of some graduate students, while simultaneously creating opportunities for others to thrive. Findings from this work underscore the importance of creating inclusive and accessible educational spaces to support graduate students’ resilience and HRQOL currently, and in times of crisis.

## Introduction

The COVID-19 pandemic has altered the lives of individuals, including post-secondary students [1–8]. In March 2020, following the World Health Organization’s (WHO) declaration of a global pandemic, the Ontario provincial government implemented public health protections that significantly impacted the lives of post-secondary students, such as stay-at-home orders that resulted in the closure of academic institutions. As such, many students were required to study from home and learn remotely during this timeframe. The ongoing COVID-19 pandemic [9] continues to be associated with challenges for students [10] and in January 2024, the WHO acknowledged that COVID-19 remains a major threat and urged member states not to dismantle established safety protocols [11]. It is not surprising then, that the COVID-19 pandemic has negatively impacted some students’ health-related quality of life (HRQOL) [1–8]—a measure of perceived mental, physical, and social wellbeing [12].

With respect to students’ mental wellbeing during the pandemic, Lorenzo and colleagues [6] conducted a longitudinal study (December 2020-January 2022) to assess changes in depression, anxiety, and stress scores over time among a cohort of post-secondary students (*N* = 1465; 33.7% graduate; 66.1% undergraduate) from Ontario, Canada. The authors concluded that participants reported mental health problems at each time point, with 52.4% exceeding the clinical cut-off scores for depression and anxiety [6]. Additionally, Ewing and colleagues [4] conducted semi-structured interviews with Ontario post-secondary (*N* = 20; first-year undergraduate) students in the Winter of 2021 to explore the psychological impacts of the COVID-19 pandemic on students with and without pre-existing mental health concerns. The authors found that post-secondary students with pre-existing mental health problems reported being able to cope more effectively with the pandemic than students without pre-existing mental health problems (Ewing et al., 2022). However, *all* students reported new and unique stressors associated with the pandemic, including worry for the health of loved ones and themselves, the unpredictability of future waves of the virus, and concerns regarding delays in degree completion [4]. Further, King and colleagues [5] investigated the impact of the COVID-19 pandemic on the mental health of three first-year undergraduate student cohorts: (1) 2018 (pre-pandemic); (2) 2019 (transitional); and (3) 2020 (during pandemic) at a university in Ontario, Canada. The authors found that all cohorts had high levels of clinically significant symptoms entering university; however, cohorts studying during the pandemic reported clinically higher levels of depression and anxiety compared to the pre-pandemic cohort [5]. Moreover, in a global meta-analysis that examined the prevalence of clinically significant depression and anxiety among post-secondary students (*N* = 1,732,456) during the early stages of the COVID-19 pandemic, the authors found that 30.6% and 28.2% of post-secondary students reported clinical levels of depression and anxiety, respectively [8].

The COVID-19 pandemic has not only impacted post-secondary students’ mental wellbeing, it has also impacted their physical and social wellbeing. Notably, in February 2021, Ahmad and Aires [1] conducted focus groups with undergraduate students (*N* = 37) at a Canadian university to ascertain the impact of the pandemic on students’ daily life, stress, and coping. Participants reported changes to their daily routine that negatively impacted their physical health and wellbeing, including a decline in physical activity, disrupted sleep, and poor dietary habits [1]. Further, participants described how the COVID-19 pandemic impacted their social connectivity, noting increased feelings of social isolation and longing for in-person classes and activities [1]. The lack of social connectedness reported by participants was not a unique experience, as it has been commonly reported by authors in the literature [2, 7]. Specifically, in July of 2020, Appleby and colleagues [2] investigated the impact of the COVID-19 pandemic on the experiences and mental health of undergraduate students (*N* = 3,013) at a university in Ontario, Canada and found that 56% of students reported feeling lonely, restless, and anxious as a result of social isolation. Participants described limited in-person interactions with peers, friends, and family which, in turn, negatively impacted their wellbeing [2]. Similarly, in March-April 2021, Morava and colleagues [7] conducted a series of focus groups with undergraduate students (*N* = 37) at an Ontario university, with the purpose of exploring how the COVID-19 pandemic was affecting students’ communities of learning. Participants described a lack of social interaction and connection, resulting in feelings of isolation for many [7]. While it is evident that the COVID-19 pandemic has been negatively impacting the HRQOL of post-secondary students, specifically with respect to their mental, physical, and social wellbeing, less is known regarding how the pandemic has been impacting graduate students more specifically.

To date, the majority of studies that have reported on the mental health and wellbeing of post-secondary students during the pandemic either focused on undergraduate students or combined cohorts of undergraduate and graduate students [1–8]. While there is value in combining cohorts (e.g., increased sample size), group differences are not typically considered, which is particularly concerning given the fact that undergraduate and graduate students respond to and experience stress differently [13, 14]. For example, in a pre-pandemic study exploring the relationship between stress and mental health, Wyatt and Oswalt [13] found that graduate students reported higher levels of stress compared to undergraduate students and were more likely to seek mental health services. Given that the COVID-19 pandemic introduced new stressors (e.g., fear of infection) [15] while exacerbating pre-existing concerns (e.g., mental health problems, social isolation, financial insecurity) [1, 16, 17], understanding the experiences of graduate students during the pandemic is particularly important given their problematic stress levels pre-pandemic [18].

The Student Experience in the Research University (SERU) Consortium survey was administered to graduate and professional students (*n* = 15,346) from May to July 2020 with the purpose of assessing graduate students’ mental health during the COVID-19 pandemic in the United States [16]. The authors concluded that 32% of graduate and professional students screened positive for depression, while 39% screened positive for anxiety [16]. Notably, the prevalence of depression was 2 times higher, and the prevalence of anxiety was 1.5 times higher among graduate and professional students in 2020 compared to 2019 [16]. Further, to explore what graduate students found to be particularly worrisome early in the pandemic, Kee [15] conducted a qualitative study with 7 participants during Spring 2020 at a college in the United States. Students reported experiencing fear and anxiety, especially regarding the uncertainty surrounding the virus, which were compounded by personal responsibilities [15]. Additionally, graduate students described coping mechanisms including setting work-life boundaries, receiving social support from online communities, as well as maintaining hope [15]. It is worth noting that historically, when navigating stressful experiences, graduate students have been found to be more likely than undergraduate students to use active coping strategies [19]; bolstering resilience is one method that can help students cope [20].

Though resilience is difficult to define due to varied conceptualizations, it can be broadly understood as a dynamic process wherein psychosocial and environmental factors interact to enable an individual to survive, grow and even thrive despite exposure to adversity [21–23]. Importantly, resilience has been found to serve as a protective factor against psychological distress during the COVID-19 pandemic [24]. In a longitudinal study conducted by Riehm and colleagues [24], the association between resilience and mental distress early in the COVID-19 pandemic (March-August 2020) was assessed among a sample of adults (*N* = 6,008) living in the United States. The authors found that those who reported low to moderate levels of resilience were more likely to experience mental distress, in comparison to those who reported high levels of resilience [24]. The relationship between mental health and resilience has also been explored among graduate students globally during the COVID-19 pandemic [25, 26]. Clark and colleagues [25] explored the association between resilience and psychological distress among health profession graduate students living in the United States during January-March 2021 of the COVID-19 pandemic. The authors concluded that higher resilience scores were associated with fewer burnout symptoms and improved overall wellbeing among graduate students [25]. Further, Oducado and colleagues [26] examined the impact of resilience on perceived stress, anxiety, and fear among graduate students (*N* = 203) in the Philippines during the COVID-19 pandemic (Summer 2020). While participants experienced moderate to high levels of stress, anxiety, and fear, resilience had a protective influence, such that it was negatively associated with stress and fear [26]. It is clear that resilience plays an important role in helping to protect graduate students against mental health problems during the COVID-19 pandemic.

It is evident, based on the above review of literature, that the COVID-19 pandemic has negatively impacted the HRQOL (mental, physical, and social wellbeing) of post-secondary students, including graduate students, in Ontario, Canada. Additionally, resilience has been found to serve as a protective factor against psychological distress among graduate students and adults globally [24]. To date, however, the lived experiences of HRQOL and resilience among *diverse* samples of graduate students during the COVID-19 pandemic remains unknown. This is particularly important as the COVID-19 pandemic has disproportionately impacted the health of marginalized groups [27–30]. Specifically, Boserup and colleagues [27] used data from the COVID-19 Tracking Project and the Centers for Disease Control and Prevention (CDC) and concluded that COVID-19 infection, death, and hospitalization rates were higher among racial/ethnic minority groups than White people in the United States. The authors also found that older age was associated with an increase in COVID-19-related deaths [27]. Similarly, in a rapid review conducted by Lebrasseur and colleagues [28], it was reported that the COVID-19 pandemic has perpetuated ageism, negative psychological symptoms, and poor physical health among older adults. In addition to racial/ethnic minorities and older adults, the COVID-19 pandemic has negatively impacted sexual and gender minority groups. Notably, Moore and colleagues [30] found that during the early stages of the COVID-19 pandemic (March-June 2020), adults in sexual and gender minority groups who were living in the United States reported increased symptoms of depression and anxiety and lower levels of social support compared to their cisgender counterparts. Parents have also been described as an equity-deserving population and in a study conducted by Kerr and colleagues [29], they reported high levels of depression and anxiety, as well as parental burnout and negative emotions such as worry and anger during the early months of the pandemic (April-May 2020). It is thus evident that the COVID-19 pandemic has disproportionately impacted equity-deserving groups including racial/ethnic and sexual and gender minorities, older adults, and parents; however, less is known about how the pandemic has impacted the HRQOL and resilience of graduate students belonging to these groups.

Of the aforementioned studies that reported on the HRQOL and/or resilience of post-secondary students in Ontario, Canada, the study populations were primarily comprised of White, cisgender students [2, 5, 6, 25, 26]. Additionally, the majority of the described studies were quantitative in nature [2, 5, 6, 8, 25, 26]. Only three studies were qualitative [1, 4, 7], and of these studies, only one reported demographic characteristics of participants [4]. This is worth noting as qualitative work lends itself to a more nuanced understanding of participants’ lived experiences [31], which is particularly important when exploring groups that are historically silenced [32]. Given that the COVID-19 pandemic has had a disproportionate impact on the health of marginalized groups such as people of colour, those of low socioeconomic status [33], sexual and gender minorities [30], and parents [29], understanding the lived experiences of graduate students who belong to these groups is critical. To our knowledge, researchers have not used purposive sampling to explore the resilience and HRQOL of a diverse sample of graduate students, making this a notable gap in the literature. To this end, and as a part of a larger mixed-methods study titled, *A* *C**ross-Sectional Examination of Ontario Graduate Students’ Levels of Resilience* *A**nd Health-**R**elated Quality of Lif**E* *During the COVID-19 Pandemic (CARE)*, the purpose of this qualitative study was to understand the lived experiences of resilience and HRQOL among a diverse sample of graduate students approximately 18 months into the COVID-19 pandemic in Ontario, Canada.

## Methods

### Study design and procedures

Ethics approval for the *CARE* study was received from the host institution’s Non-Medical Research Ethics Board (NMREB #119239). Participants were recruited for the study through the host institution’s mass emailer system and social media platforms (i.e., Twitter, Instagram, Facebook, LinkedIn). To be eligible for the study, individuals needed to be enrolled as a full-time graduate student at an Ontario university and be able to read, write, and speak English. Interested participants were asked to click the link on the study advertisements, which brought them to a Qualtrics^XM^ survey (Qualtrics, Provo, UT) that included the letter of information, eligibility and consent process, as well as the questionnaires a more fulsome account of the quantitative methods can be found in Shillington et al. [34]. At the end of the survey, participants were redirected to a new link where they were invited to submit their name and email address if they were interested in participating in a follow-up interview.

The semi-structured interviews were intended to build from the previous findings reported in Shillington et al. [34], by capturing participants’ lived experiences of resilience and HRQOL during the COVID-19 pandemic. Given the variability in graduate student demographics, purposive sampling based on participant demographic criteria was used to obtain a diverse sample of interview participants (per the guidance of Lavrakas [35]). To achieve a diverse sample, research assistants compiled the demographics of students who expressed interest in participating in an interview. Next, a sample of graduate students were purposefully selected to represent diversity in age, gender, ethnicity, parental status, university, degree, and faculty. Additionally, students from different universities, degrees (e.g., masters, doctoral, professional), and faculties were considered. Those who were invited to participate in an interview were given the letter of information and asked to provide their availability. The number of interviews conducted was based on the work of Guest and colleagues [36], who suggested that theoretical saturation was likely to be achieved by the 12th interview. As such, 14 interviews were conducted with an additional two serving to ensure saturation had, in fact, been reached. At the beginning of each interview, participants were asked to provide verbal consent and, in an effort to diminish social desirability bias, were reminded that there were no right or wrong answers and they could refuse to answer any questions [37]. To support data credibility [38], the interviewer member checked between questions by summarizing participant responses and reflecting back. The interviews took place via Zoom (*n* = 13) or the telephone (*n* = 1) with a trained interviewer (KS) and ranged from 60–90 minutes in length. All interviews were transcribed verbatim by a member of the research team.

### Positionality and epistemological approach

All authors share the epistemological and ontological position of post-positivism. In sharing this stance, the authors believe that researchers should serve as independent observers of the social world, such that social realities should be understood from the perspective of the participant, rather than the researcher [39]. The authors acknowledge that participants are “active subjects who are productive of their social reality, not simply the objects of social forces” [39, p. 3]. Accordingly, the authors recognize their own perspectives and biases as they interact with the social worlds they are researching and acknowledge that they impose a second level of interpretation during data collection and analysis [39]. Further, given the nature of the current study and in the spirit of self-reflexivity, it is worth noting that the authors identify as White women, who have completed post-graduate degrees. At the time the research was conducted, the lead author was a graduate student, similar to participants in the study. The authors acknowledge that they might not share the same lived experiences of graduate students of diverse backgrounds; however, all authors interact with and have a vested interest in the wellbeing of graduate students. To this end, it is possible that the authors’ positionality influenced the research process.

### The COVID-19 pandemic context during data collection

Prior to data collection for the present study, which occurred March 14–29, 2022, Ontario had returned to a modified Step Two of the Roadmap to Reopen framework due to rapid spread of the COVID-19 Omicron variant [40]. This resulted in the closure of indoor dining and gyms, limits on indoor and outdoor gatherings, lower capacities at retail stores and malls, and the closure of schools and in-person learning [40]. By March 2022, schools had reopened, in-person learning resumed, and masks were no longer mandated in education settings [41].

### Data collection

The interviewer (KS) followed a semi-structured interview guide (S1 Appendix) that asked participants to share their views, insights, and reflections about the previously collected quantitative findings from the *CARE* study [34]. Specifically, the interviewer shared findings from the previously validated Connor-Davidson Resilience Scale-10 (measuring resilience) [42] and the RAND 36-Item Health Survey 1.0 (measuring HRQOL) [43], by verbally providing the average scores and their interpretations. The interviewer then asked participants to discuss the extent to which the findings resonated or did not resonate with them by providing additional details and examples relative to their lived experiences. While the interview data were collected in March 2022, participants were asked to reflect back on the timeframe that quantitative data was collected (August-September 2021). Additionally, to expand our understanding of students’ experiences of resilience conceptualized by Rossouw and Rossouw [44], participants were asked to describe what supported and undermined their resilience with respect to: (1) vision; (2) composure; (3) tenacity; (4) reasoning; (5) collaboration; and (6) health. When prompted, the interviewer provided examples of each domain to aid in participants’ understanding. Further, participants were asked about challenges/barriers they faced to being resilient, as well as what contributed to their inner strength.

Data collection and analysis were guided by principles of credibility, dependability, confirmability, and transferability [38] (Table 1). Additionally, to support the credibility of the data and minimize bias, the principal investigator served as a peer debriefer during data collection and analysis [38]. This involved the lead author and principal investigator meeting throughout the data collection stage and before analysis to explore the lead author’s personal values and perspectives that had the potential of biasing the interpretation of the data [45]. The principal investigator posed questions to aid the lead author in understanding her positionality in the inquiry [38]. Following analysis, the lead author and principal investigator reconvened to ensure the themes and associated quotes had minimal bias.

**Table 1:** Measures taken to ensure data trustworthiness.

**Credibility**	The interviewer member checked between questions, as well as at the end of each interview by summarizing and reflecting back participant responses to ensure that the researcher understood and interpreted participants’ responses correctly. To minimize bias, the interviewer consulted with a peer debriefer throughout data collection and analysis. Biases were identified, recorded, and considered to ensure the analysis was not influenced by researcher bias.
**Dependability**	During the interviews, the researcher took detailed reflexive notes. Throughout data collection, the interviewer met with the principal investigator to debrief the interviews. Additionally, the interviewer documented the methods and study procedures in detail for the purpose of an audit trail.
**Confirmability**	Four researchers conducted thematic analysis. The researchers independently and simultaneously read the transcripts and documented notes to create the coding structure, inclusive of themes and their associated descriptions. This was an iterative, multi-step process resulting in a finalized codebook that all researchers agreed upon. Following data analysis, the interviewer met with the peer debriefer, who familiarized themself with the data, themes, and supporting quotes, to address any conflicting biases.
**Transferability**	To determine whether the findings from the current study are transferable to other settings, the lead author provided a thick, rich description of the research process and procedures.

### Data analysis

Interview data were analyzed by question (i.e., deductively) using an inductive content analysis approach [46], following the method for thematic analysis outlined by Braun and Clarke [47]. First, the lead author who conducted the interviews (KS) and the principal investigator (JI) met to discuss and create a preliminary codebook, based on commonalities that emerged during the interview process, as well as reflexive notes from the interviews [47]. The notes generated contributed to data triangulation, a method used to ensure credibility [38, 47]. The initial codebook consisted of preliminary themes and associated descriptions. Next, random coding dyads were generated and consisted of the lead author (KS) and three research assistants (JC; JB; ZR), who were each initially assigned 5 transcripts (representing 10 out of 14 transcripts) to analyze using the preliminary coding structure. The use of multiple coders was to support confirmability [38]. The researchers then independently and simultaneously familiarized themselves with the data by reading and re-reading the transcripts while making notes regarding the codebook and associated themes and descriptions [47]. During this process, the researchers generated initial codes and then collated the codes into existing, modified, and/or new themes [47]. Following this process, the dyads met individually and then with the larger group to discuss inconsistencies, resolve disagreements, and bring forward any new perspectives that emerged during their read throughs [47]. This was an iterative process that involved going through the preliminary codebook (i.e., each theme and associated description) and providing space for members of the coding team to challenge the initial themes; this process continued until consensus was reached among the group [47]. As a result, refinements were made to the codebook, inclusive of the themes and working definitions [47]. Lastly, the researchers reviewed the agreed upon themes by checking to ensure that they related to the coded extracts (step 1) and then to the entire dataset (step 2) [47]. Finally, the researchers defined and named the themes to be included in the analysis [47]. Once the researchers agreed upon the coding structure, each coder was assigned 7 transcripts (i.e., two coders per transcript). The coding team then independently analyzed their assigned transcripts using the finalized codebook [47]. Upon completion, the lead author merged the analysis outputs into one report. In an effort to enable researchers to replicate the study and to support dependability [38], the methods and study procedures were documented in detail.

## Findings

Of the 376 participants who completed the *CARE* survey, 151 expressed interest in a follow-up interview, and 14 completed a semi-structured interview. The average age of interview participants was 30.3 years (*SD* = 9.4), though participants ranged in age from 23 to 58 years old. One fifth of interview participants identified as non-binary (21.4%), 28.6% identified as male, and 50.0% identified as female. The majority of participants identified as non-European origins including Asian (14.3%), other North American (14.3%), Caribbean (7.1%), Latin, Central, and South American (7.1%), African (7.1%), Middle Eastern (7.1%), and mixed origins (7.1%). All participants reported residing off-campus (100%), and the majority lived in London, Ontario (57.1%) and attended Western University (85.7%). Most participants reported being single (64.3%), while some participants reported living with parents/guardians (28.6%), a spouse/partner (37.5%), friends/roommates (21.4%), children (21.4%), and/or siblings (14.3%). Additionally, 14.3% of participants reported being the primary caregiver for an individual with a health condition/disability. The majority ***of*** participants were employed (85.7%) and pursuing a Master’s (42.9%) or Doctoral degree (50%). Full demographic details of interview participants can be found in Table 2, while full demographic details for all participants can be found in Shillington and colleagues [34].

**Table 2:** Demographic information of interview participants.

Participant Characteristics (*n* = 14)^1^		
**Age (years)** ^**2**^	***M* (SD)**	
Total	30.3 (9.4)	
	***n***	**%**
**Sex**		
Female	9	64.3
Male	5	35.7
**Gender**		
Female	7	50.0
Male	4	28.6
Non-binary	3	21.4
**Lived experience as trans person**		
Yes	2	14.3
No	12	85.7
**Ethnicity**		
North American Aboriginal origins	0	0
Other North American origins	2	14.3
European origins	5	35.7
Caribbean origins	1	7.1
Latin, Central, and South American origins	1	7.1
African origins	1	7.1
Asian origins	2	14.3
Mixed origins/Multiracial	1	7.1
Middle Eastern origins	1	7.1
**Geographic location** ^**3**^		
London, Ontario, Canada	8	57.1
Other Cities in Ontario, Canada	6	42.6
**Residence**		
On campus	0	0
Off campus	14	100
**Living situation (members in household)** ^**4**^		
Parent(s)/guardian(s)	4	28.6
Spouse/partner	5	37.5
Friend(s)/roommate(s)	3	21.4
Child(ren)^**5**^	3	21.4
Sibling(s)	2	14.3
Relative(s)	0	0
Alone	2	14.3
**Primary caregiver for individual(s) with health condition/disability**		
Primary caregiver	2	14.3
Not a primary caregiver	12	85.7
**Marital status**		
Single	9	64.3
Married/common law/engaged	5	35.7
Divorced/separated	0	0
**University**		
Western University	12	85.7
Other Ontario Universities	2	14.2
**Faculty**		
Health Sciences, Public Health, and Kinesiology	4	28.6
Arts and Social Science	2	14.3
Arts	2	14.3
Education	1	7.1
Social Science and Humanities	3	21.4
Nursing	1	7.1
Science	1	7.1
**Degree**		
Master’s	6	42.9
Doctoral	7	50.0
Professional	1	7.1
Combined	0	0
**Employment status**		
Employed	12	85.7
Unemployed	2	14.3
**Income**		
< $25,000	3	21.4
$25,000 - $50,000	3	21.4
$50,000 - $75,000	2	14.3
$75,000 - $110,000	0	0
$100,000 - $150,000	1	7.1
$150,000- $200,000	2	14.3
> $200,000	1	7.1
I prefer not to answer	2	14.3

^1^The total sample size was 14 participants; not all categories summed to equal the total sample due to missing data.

^2^Age was collected as a continuous variable.

^3^To maintain participant anonymity, university institutions and geographic locations with only one participant were amalgamated accordingly.

^4^Due to the nature of how the question was asked (e.g., select all that apply), it is possible that participants are included in multiple categories under ‘Living Situation’.

^5^Data on parental status was not explicitly collected; however, participants were asked to report the members in their household (inclusive of children). It is possible that there were participants in the current study who identified as a parent, but did not live with their children.

Five themes emerged from the data: (1) cultural influences on resilience; (2) the role of privilege/power in shaping resilience; (3) how life stage and past experiences support resilience; (4) how the COVID-19 pandemic has undermined the resilience of equity-deserving groups; and (5) the role of disability/chronic pain. In the subsequent paragraphs, themes are presented within each broader research question category (i.e., resilience and health-related quality of life) along with illustrative quotes. Although quotes may be relevant to more than one theme, they are presented in the theme in which the quote was deemed to best fit.

### Resilience

#### Cultural influences on resilience

Some participants in the current study described how culture can influence a person’s resilience. For one participant (N05), being an international student was “proof” of their resilience, saying:

I [do] consider myself a resilient person… [I’m] going back to the fact of being an international student, but I believe that if you’re going through the entire process of moving to another country, understanding a total[ly] different culture… communicating in a different language, or things like that… That’s a really strong proof of resilience.

Another participant (N02) identified as being an Occupational Therapy student wherein their program was comprised of students with cultural similarities, saying:

Maybe another thing to add about our program is that I know… they really don’t accept international students. So pretty much everyone is from Canada and a lot of people are from Ontario… A lot of people have their own friend groups locally…as well as the friend groups within the program. And then there’s a lot of cultural similarities… We’re all around the same age, so it’s very easy to get along with each other… I know in other graduate programs there is probably a lot more international students who might feel a lot more isolated or not be able to fit in quite as easily.

Interestingly, one participant described feeling as though they were from a different culture due to their age. This participant (N09) said, “It’s almost like a cultural difference. It’s almost like age is like a… I’m being… perceived as if I’m from a different culture….” Another participant (N12) underscored how the practice of meditation and mindfulness has a cultural importance to their resilience saying, “…a dedicated meditation and mindfulness practice and yoga practice… is very important for myself in a cultural sense….” Further, one participant (N08) highlighted the intersection of culture and inclusivity to support their resilience saying, “Being around people who are like-minded is really important, and being in spaces that are community-based is really important. So be that, a queer community space or… I volunteered for a queer library for a while and that was fantastic….” Thus, even though participants’ interpretations and understanding of culture differed, they described how it influenced their resilience in various ways.

#### The role of privilege/power in shaping resilience

Many participants underscored privilege as a factor that supported their resilience, especially with regard to having financial freedom. This was emphasized by one individual (N02) who said:

I am in a very lucky… more privileged position, in that my parents are supporting the cost of my degree. So, I don’t have to work during the school year, which is a big benefit for me, and which I [can] see is very draining for some of my classmates. And I’m assuming to be very draining for a lot of other grad students who aren’t as lucky or in as comfortable of a position.

Similarly, another participant (N08) described how being open to opportunities can support resilience while acknowledging that not everyone is given equal opportunity, saying:

I think as much as one door closes, another will open, and you need to just look for that opportunity. And again, I am aware that this comes with a little bit of privilege to say that. If you’re in a situation where you are living pay cheque to pay cheque, this is probably a lot less true. But for somebody who has the privilege that I do, opportunities will present themselves to you if you take them….

Additionally, one individual (N09) reflected on the increased ease to be resilient if you have financial stability saying, “I had a good childhood, good family, good income. And those things have made it so that it’s easier, I think, to carry on. If you’re not… incredibly worried about finances or… the basics, then… maybe you can be more resilient.” Another individual (N07) highlighted how the COVID-19 pandemic helped to shed light on inequities and the role that privilege plays in shaping resilience saying:

I think that I’m quite fortunate… I realize that I have a lot of privilege that comes… with my financial stability, having a supportive partner, with my ethnic background… I realize and identify that as we’ve gone through the pandemic, we’ve certainly brought a lot more awareness to racism and discrimination and how that affects our health and our position, our susceptibility to infectious disease. So, I think I’ve become more aware of the privileges that I do have because it would not be the same for me if I had a different skin colour or different economic status, right? If I didn’t have the job that I have with the support that I have… Some of which comes from hard work, investment in my education early on when others in my same life stage weren’t, but some of it is just pure luck as well, right? In the up-bringing I had, the family I was born into, the country I was born in, for example… So, I think that’s important to identify….

Thus, while many participants identified as being resilient, they also acknowledged how privilege played a role in their resilience. Conversely, some students described an unbalanced power dynamic in academia, which negatively impacted their resilience.

This was emphasized by one participant (N12) who said:

I think it just stems from norms in academia in any case, right? Which is like, you’re not to speak truth to power [chuckles], you’re not to really question how things are set up… I would argue that a lot of people our age *do* see that academic norms are quite harmful and they’re like, ‘[It] seems very unnecessary, why don’t we do it differently?’ But then for profs… who have made their careers in those spaces, they kind of become agents of reproducing those kind of norms, even though they’ve… been negatively affected by them [the norms] themselves.

Similarly, another participant (N03) highlighted challenges within the student-supervisor dynamic, specific to the COVID-19 pandemic, saying:

It felt like there was kind of a lack of… empathy, or just [a lack of] understanding of where we were coming from… It’s a little bit challenging to be resilient when it feels like the person on the other side doesn’t necessarily understand… like what you’re going through. Because it is a unique experience to be trying to do grad school, especially while there’s a pandemic going on. And so, I think when there’s that miscommunication or lack of understanding, it becomes a little bit more difficult to be resilient because it doesn’t feel like you’re necessarily supported the way that you want to be.

#### How life stage and past experiences support resilience

Participants described factors that supported their resilience, including the stage of life they were in and learning from past experiences. Even though many participants described going through difficult times, they spoke to how those experiences helped to strengthen their resilience, making participants more equipped to deal with future hardships. This was emphasized by one participant (N04) who reflected on how age can impact resilience, by describing the difference between going through challenging life experiences when they were young compared to now, saying:

I guess having been resilient from a young age has helped me to understand now that I can deal with things a lot better than I thought I could… I have a self-awareness that we’ve established comes from resilience. I’ve been able to, again, ask for those supports… [and] identify or learn skills to put into my toolbox… So, learning new skills to self-regulate or to cope… maybe healthier or more productive ways of coping than the ways I used when I was having to be resilient from the beginning, without the help.

Another participant (N10) echoed this sentiment as they “considered [themself to be] a highly resilient person given several [life] experiences.” Many participants perceived their resilience to be high because of having gone through challenging experiences, as highlighted by one participant (N11) who said:

I feel like my perception of my own resilience is higher now because… I have the hindsight of thinking, ‘Oh, I’ve succeeded in these difficult situations and come out unscathed mostly so… Clearly, I can do it,’ which I feel like makes it easier to be optimistic moving forward about resilience.

Reflecting on previous life experiences to overcome current challenges was commonly reported by individuals, as another participant (N12) emphasized the importance of “remembering that you can go through some incredibly, incredibly difficult times… but at the end of the day… your failures are not going to be as high stakes as they feel at the time.” Additionally, one participant (N07) highlighted how their life stage played a role in their resilience saying:

I don’t know the average age of graduate students, but I think I’m probably a bit older. I’m knocking on the door of the fourth decade of my life… I had a big space between my master’s and my doctorate and so I think… learning through that probably helps me, for sure… and knowing that sometimes it takes experience, practice, and just hard work….

For this participant, being of older age and having more life experience contributed to their resilience.

#### How the COVID-19 pandemic has undermined the resilience of equity-deserving groups

Participants described how the COVID-19 pandemic, inclusive of the protections implemented and the associated uncertainty, has undermined the resilience of equity-deserving groups including parents and immunocompromised and disabled people. Notably, one individual (N07) discussed the challenge of balancing evolving information, anxieties, and parenting responsibilities, saying:

Of course, at the beginning of the pandemic there was a lot of unknowns and anxiety and concerns, especially clinically with the evolving information… So… it’s just a bit challenging to balance, especially [with]…school age children who I don’t have childcare [for] because of COVID.

Other individuals underscored how the length of the pandemic has undermined their resilience, including the lack of support for immunocompromised and disabled people. For example, one participant (N03) said:

The longer things [the pandemic has gone] on… the less resilient I felt… Initially… you don’t really know how long things will be the way they are and so… the hope aspect plays into it… It’s easier to… be resilient and… buckle up and go along for the ride. But I think the longer things continue to go on, it just felt a little bit more challenging.

Another participant (N08) described the pandemic as “eye-opening” regarding a lack of support for at-risk groups saying, “It’s been really eye-opening to see how many people are not in support of immunocompromised or disabled people and are not willing to wear masks for [those] groups… It’s a little shocking….” Whether it was managing multiple roles, the length of the pandemic, or the lack of compassion for at-risk groups, it is evident that the COVID-19 pandemic has undermined the resilience of equity-deserving people in the current study.

### Health-related quality of life

#### The role of disability/chronic pain

Participants reflected upon how having chronic pain and/or a disability might impact an individual’s role as a graduate student. For example, one individual (N07) emphasized that ill health does not always manifest as visible symptoms saying, “It’s hard to know… other people’s personal health, especially when you just… see them in class and… that might not represent their health… you know? Maybe they have a big chronic disease, and they appear fine in class, but they struggle outside of that….” Another individual (N01), while reflecting on the challenges they experienced during a time when they had broken their foot and that “[i]f I had a longer-term disability, I could see how that would be impactful.” Other participants who identified as having a disability described how it impacted their day-to-day as a graduate student. One individual (N11) with a physical disorder described changing limitation experiences based on their medical condition, saying, “I find most of the time I don’t have any limitations, and then some of the time… I have different flare-up issues, and on those particular days… I do have issues.” Another participant described a history of knee pain, having undergone four knee surgeries. For this individual, the lockdown period of the COVID-19 pandemic has had a negative impact on their disability as they were not able to rely on certain exercises/activities at the gym, due to gym closures, to manage their pain (N06). In contrast, one participant (N08) emphasized how the COVID-19 pandemic has increased accessibility, which in turn improved their academic performance. Pre-pandemic, this individual (N08) described regularly missing class due to health issues, which meant they were “always running to catch up with everybody else” instead of being on track with the rest of the class. Thus, “having [the] opportunity to actually attend every single class [remotely]… radically changed [their] performance”, such that they were able to “perform to [their] highest capabilities as a student.” Additionally, this participant (N08) emphasized:

There’s been such a… disparate experience of the pandemic based on disability, be that mental health or a physical disability… I feel like so many of the benefits that were brought to me by the pandemic by having classes online, have extremely negatively impacted other people. Like the average take on online classes is [a] strong hatred, strong dislike [by some other students], right? And I’m over here like ‘No, this is the best class I’ve ever had!’ …I do think that accessibility is a really major thing that has come [out] of [the] pandemic and I think that the binary distinction has really increased between the two because of things like online classes. So yeah, I think… [the] disabled experience is very different sometimes.

Interestingly, another participant (N04) suggested that “…the people who, because of the pandemic, are thriving in the online learning environment or in the hybrid environment, have seen an increase in their health.” Therefore, while few participants in the current study identified as having a disability, it is important to highlight their experiences to provide adequate support, especially because the COVID-19 pandemic has improved accessibility in educational settings.

## Discussion

The purpose of this qualitative study was to understand the lived experiences of resilience and HRQOL among a diverse sample of graduate students approximately 18 months into the COVID-19 pandemic in Ontario, Canada. Participants described how culture, life stage, and past experiences can influence resilience, the role of privilege/power in shaping resilience, and ways in which the COVID-19 pandemic has undermined the resilience of equity-deserving groups. Additionally, participants underscored the role of disability and chronic pain as they related to daily living during the pandemic.

Though the understanding and conceptualization of culture differed among participants, many underscored the ways in which culture intersected with resilience. For one participant, being an international student was “proof” of their resilience, as they experienced unique stressors including moving to another country and learning a new culture and language. This finding aligns with work conducted by Larcombe and colleagues [48], who suggested that international students are relatively resilient and are able to cope with the unique stressors they encounter. Specifically, the authors concluded that international students in their study reported higher levels of self-compassion than domestic students, suggesting that self-compassion might buffer against the stressors that international students encounter and foster resilience [48]. This said, Larcombe and colleagues [48, 49] also recommended enhancing environmental psychosocial resources such as social connections and belonging, as these resources have been found to predict student wellbeing. This recommendation aligns with findings from the current study, as one participant emphasized how being a part of “like-minded” community-based groups, such as queer community spaces, can support resilience. In line with this finding, Abreu and colleagues [50] explored how community and cultural values have informed the experiences of lesbian, gay, bisexual, transgender, and queer (LGBTQ) people (*N* = 130) during the COVID-19 pandemic (May-June 2020). The authors concluded that participants relied on intergenerational resilience passed down from their ancestors in the LGBTQ community to help them navigate the COVID-19 pandemic [50].

In addition to culture, life stage and past experiences were identified by participants as factors that can influence resilience. Specifically, some participants identified that being of older age supported their resilience. This finding is not surprising, as Hayman and colleagues [51] concluded that late life “is characterised by a unique balance between losses, associated with vulnerability and [social, psychological, and attitudinal] resource restrictions, and potential gains based upon wisdom, experience, autonomy and accumulated systems of support, providing a specific context for the expression of resilience” (p. 577). The authors also suggested that resilience may be higher in older adults compared to other ages, as a result of past life experiences [51]. This aligns with findings from the current study, as many participants perceived their resilience to be high as a result of having gone through challenging life experiences. This is not surprising, as some evidence suggests that while experiencing adverse life events can have short-term negative impacts, it can also lead to increased resilience in the long-term [52]. Specifically, in a report by Seery [52], the author concluded that individuals with some prior lifetime adversity were less likely to be negatively affected by recent adversity compared to those with no history of adversity or high lifetime adversity. Thus, it is not surprising that participants in the current study attributed their resilience to having experienced difficult life experiences.

Graduate students in the current study also identified privilege as a factor that contributed to resilience. The relationship between privilege and resilience is largely unknown in the literature; however, lay literature suggests that people tend to assume members of marginalized groups are resilient because of their identities [53]. Interestingly, some students in our study suggested that it might be easier to be resilient because of their privileges (e.g., economic status, race), providing a differing perspective than the aforementioned discourse. It is worth noting that while 64% of study participants identified as a person of colour, more research is needed to explore the relationship between resilience and privilege. Importantly, researchers argue that resilience should be revisited and redefined in light of associated inherent biases and racism, as the field is dominated by White middle-class voices [54]. In contrast to privilege, some participants in the current study discussed how the norms of academia can be harmful, including power imbalances and a lack of empathy from supervisors. These findings align with what is reported by Cho and Hayter [18] in their systematic review (2000–2018; *N* = 34 studies) on the impact of stress among graduate students. Specifically, these authors concluded that the academic community and environment can either positively or negatively impact student stress [18]. Common sources of stress among graduate students included academic demands, funding sources, individual circumstances, and level of support from peers and advisors [18]. Notably, Cho and Hayter [18] concluded that graduate students who experienced positive advising, mentoring, and support tended to experience less stress.

While participants described several factors that supported their resilience, they also highlighted factors that undermined their resilience; most notably, the COVID-19 pandemic. Participants underscored how the COVID-19 pandemic has disproportionately impacted equity-deserving groups including parents and individuals who are immunocompromised and/or disabled. Notably, one participant underscored difficulty balancing the unknowns of the pandemic, evolving information, and parenting school-aged children without childcare. This finding aligns with previous qualitative work, as participants in a study conducted by Obeng and colleagues [55] emphasized that the early months of the pandemic were particularly stressful for parents who had to work and homeschool their children simultaneously during the week. Similarly, Petts and colleagues [56] examined whether loss of childcare and new homeschooling demands were associated with employment outcomes for parents during the early months of the COVID-19 pandemic (April 2020). For parents of school-aged children, the authors concluded that homeschooling was associated with adverse employment outcomes among mothers, but not fathers [56]. This is particularly concerning given that 50% of participants in the current study identified as female and 21% reported that children were members of their households. Parents are not the only group that have been disproportionately affected by the COVID-19 pandemic, as a participant in the current study also highlighted a lack of support towards immunocompromised and disabled people. This finding aligns with work conducted by Rechner and colleagues [57], who conducted a qualitative study with individuals with disabilities and their caregivers, service providers, and elected governmental representatives in Delaware, United States. Participants in this study (*N* = 25) emphasized how the disability community has been overlooked in response to the COVID-19 pandemic. Additionally, schools provide one-on-one support for children with disabilities similar to the support provided by day programs for adults with disabilities; however, much of this support was lost as a result of school and business closures during the early months of the pandemic [57]. This resulted in additional responsibilities for caregivers during the day and changes in daily routines, a finding that is particularly noteworthy given that 14% of participants in the current study identified as a primary caregiver for individuals with a health condition/disability.

Some participants in the current study described the role of disability/chronic pain in relation to their identity as a graduate student. Notably, participants who did not overtly identify as having a disability/chronic pain empathized with those who did, while others described varied lived experiences. Specifically, one student underscored how the pandemic created barriers for them, while another highlighted how the pandemic improved accessibility. This is one of the first studies to shed light on the lived experiences of disability/chronic pain among students during the COVID-19 pandemic; however, in a pre-pandemic study where Hamilton and colleagues [58] explored the experiences of students (*N* = 77) with chronic illnesses in the United Kingdom, participants described feeling undervalued because the university did not prioritize disability-related needs and accessibility. In contrast, a participant in the current study emphasized how the COVID-19 pandemic “levelled the playing field” and improved accessibility by shifting to an online/hybrid learning format. Benson [59] argues that hybrid learning is an example of “universal design”, which has been recommended by researchers in the education sector as an inclusive approach to engage students with disabilities in the learning process [60]. Thus, while the dominate discourse in the literature highlights the disadvantages of online learning (e.g., lack of interaction, technical difficulties, increased screen time), as well as students’ desire to return in-person [1], consideration needs to be given to students with disabilities and chronic illnesses; this study is one of the first to shed light on this perspective.

## Limitations

This paper is not without limitations. First, while the authors used purposive sampling to obtain a diverse sample of graduate students, they were limited to participants who completed the *CARE* survey and expressed interest in interview participation. Thus, while a notable strength of the study is the diverse population, it is worth noting that half of the participants identified as female and over a quarter were of European origins. In the future, researchers interested in conducting research with minority populations may wish to specifically target ethnic and gender diverse groups. Second, the interview guide was informed by the quantitative data from the larger study [34], which might have influenced participants’ responses, as students were asked the extent to which they resonated with study findings. This said, to mitigate social desirability bias, participants were reminded at the start of each interview that there were no right or wrong answers and that the research team was only interested in what was true for them. Further, given that the interview guide was informed by the quantitative results, the time between quantitative and qualitative data collection was a limitation. Specifically, quantitative data collection occurred between August and September 2021; however, to allow time for analysis and interview scheduling, qualitative data were not collected until March 2022. Thus, when asked the extent to which they resonated with study findings, participants had to reflect back to the time of quantitative data collection which, given the evolving nature of the COVID-19 pandemic, was a challenge. In the future, researchers may wish to shorten the timeframe between quantitative and qualitative data collection or develop an interview guide that is not informed by quantitative data.

## Conclusion

The purpose of this qualitative paper was to understand the lived experiences of resilience and HRQOL among a diverse sample of graduate students during the COVID-19 pandemic in Ontario, Canada. Findings from this study underscore factors that support and undermine graduate students’ resilience, as well as how the pandemic impacted the health of participants. Notably, this paper shed light on the role of disability and chronic pain as they related to daily living during the pandemic. This work presents a unique dichotomy between how the COVID-19 pandemic disrupted the lives of some graduate students, while simultaneously creating opportunities for others to thrive. Findings from this study underscore the importance of creating inclusive and accessible educational spaces to support graduate students’ resilience and HRQOL. Researchers may wish to build off these study findings by exploring the relationship between privilege and resilience, as well as investigating students with disabilities’ perceptions of the online learning environment, and how it might have changed throughout the ongoing COVID-19 pandemic.

## Supporting information

S1 Appendix (DOCX)
