# Supplementary material for: “I do think that accessibility is a really major thing that has come [out] of [the] pandemic”: The lived experiences of resilience and health-related quality of life among a diverse sample of graduate students during the COVID-19 pandemic
Source: PLoS One. 2024 Aug 20;19(8):e0309171. doi: 10.1371/journal.pone.0309171 (PMC11335158; doi:10.1371/journal.pone.0309171)
Supplement: S1 Appendix — (DOCX) [file pone.0309171.s001.docx]

**Appendix A**

**Interview Questions**

1. As you know, the focus of this research is on graduate students’ resilience and health-related quality of life. To start off the interview, I’m curious how you, a graduate student during this time, are doing?

*Resilience*

*As a reminder, resilience can be understood as a dynamic process in which psychosocial and environmental factors interact to enable an individual to survive, grow and even thrive despite exposure to adversity.*

1. In the baseline survey we asked a series of questions pertaining to your **resilience**. Data analysis revealed that participants’ average score was 26.04 on a scale ranging from 0-40. This means that the resilience of graduate students in our study during the months of July-August 2021 was in the lowest quartile (range = 0-29).
   1. I’m curious, what about this finding resonates with you? Or perhaps doesn’t resonate with you?
   2. What would you like to add to this?
2. What helps to support your resilience?
   1. In terms of achieving your goals/purpose?
   2. In terms of regulating your emotions (e.g., remaining calm and in control)?
   3. In terms of problem solving?
   4. In terms of your health (e.g., sleep, exercise, nutrition)?
   5. In terms of persistence and bouncing back?
3. What undermines your resilience?
   1. In terms of achieving your goals/purpose?
   2. In terms of regulating your emotions (e.g., remaining calm and in control)?
   3. In terms of problem solving?
   4. In terms of your health (e.g., sleep, exercise, nutrition)?
   5. In terms of persistence and bouncing back?
4. What are some challenges/barriers that you have faced to being resilient?
5. What has contributed to your inner strength?

*Health-Related Quality of Life (HRQOL)*

1. In the baseline survey we also asked a series of questions pertaining to your **HRQOL**, which includes eight domains. Data analysis revealed that participants’ average score for their **physical functioning** was 91.63 on a scale ranging from 0-100. This means that the physical functioning of graduate students in our study during the months of July-August 2021 was high.
   1. I’m curious, what about this finding resonates with you? Or perhaps doesn’t resonate with you?
   2. What would you like to add to this?
2. When we consider graduate students’ **role limitations due to physical health problems**, which can be understood as problems with your work or other regular daily activities as a result of your physical health, data analysis revealed that participants’ average score was 76.22. This means that the role limitations due to physical health problems of graduate students in our study during the months of July-August 2021 was relatively high.
   1. I’m curious, what about this finding resonates with you? Or perhaps doesn’t resonate with you?
   2. What would you like to add to this?
3. When we consider graduate students’ **role limitations due to emotional problems**, which can be understood as problems with your work or other regular daily activities as a result of any emotional problems, data analysis revealed that participants’ average score was 37.61. This means that the role limitations due to emotional problems of graduate students in our study during the months of July-August 2021 was relatively low.
   1. I’m curious, what about this finding resonates with you? Or perhaps doesn’t resonate with you?
   2. What would you like to add to this?
4. When we consider graduate students’ **energy/fatigue**, data analysis revealed that participants’ average score was 38.86. This means that the energy/fatigue of graduate students in our study during the months of July-August 2021was relatively low.
   1. I’m curious, what about this finding resonates with you? Or perhaps doesn’t resonate with you?
   2. What would you like to add to this?
5. When we consider graduate students’ **emotional wellbeing**, data analysis revealed that participants’ average score was 55.91. This means that the emotional wellbeing of graduate students in our study during the months of July-August 2021 was moderate.
   1. I’m curious, what about this finding resonates with you? Or perhaps doesn’t resonate with you?
   2. What would you like to add to this?
6. When we consider graduate students’ **social functioning**, data analysis revealed that participants’ average score was 67.51. This means that the social functioning of graduate students in our study during the months of July-August 2021 was moderate.
   1. I’m curious, what about this finding resonates with you? Or perhaps doesn’t resonate with you?
   2. What would you like to add to this?
7. When we consider graduate students’ **bodily pain**, data analysis revealed that participants’ average score was 76.97. This means that the bodily pain of graduate students in our study during the months of July-August 2021 was relatively high.
   1. I’m curious, what about this finding resonates with you? Or perhaps doesn’t resonate with you?
   2. What would you like to add to this?
8. When we consider graduate students’ **general health perceptions**, data analysis revealed that participants’ average score was 63.75. This means that the general health of graduate students in our study during the months of July-August 2021 was moderate.
   1. I’m curious, what about this finding resonates with you? Or perhaps doesn’t resonate with you?
   2. What would you like to add to this?
9. When we consider graduate students’ **perceived change in health**, data analysis revealed that participants’ average score was 49.22. This means that the general health of graduate students in our study during the months of July-August 2021 was moderate.
   1. I’m curious, what about this finding resonates with you? Or perhaps doesn’t resonate with you?
   2. What would you like to add to this?
